# Multicenter randomized clinical trial comparing dexamethasone versus placebo in preventing upper airway obstruction after extubation in critically ill children

**DOI:** 10.1038/s41598-022-08178-0

**Published:** 2022-03-14

**Authors:** Laura Butragueño-Laiseca, Gema Manrique Martín, Rafael González Cortés, Corsino Rey Galán, Zuriñe Martínez de Compañón Martínez de Marigorta, Javier Gil Antón, Antonio Rodríguez Núñez, Cecilia M Fernández-Llamazares, Silvia Manrique-Rodríguez, Jesús López-Herce Cid

**Affiliations:** 1 Service of Pediatric Intensive Care, Gregorio Marañón General University Hospital, Calle Doctor Castelo 47, 28009, Madrid, Spain; 2 Gregorio Marañón Health Research Institute, Madrid, Spain; 3 Mother and Child Health Research Network (Red SAMID), Madrid, Spain; 4 Department of Public Health and Pediatrics, Complutense University of Madrid, Madrid, Spain; 5 Pediatric Intensive Care Unit, Central Hospital of Asturias, Oviedo, Spain; 6 Pediatric Intensive Care Unit, Vall d’Hebron University Hospital, Barcelona, Spain; 7 Pediatric Intensive Care Unit, Cruces University Hospital, Barakaldo, Spain; 8 Pediatric Intensive Care Unit, University Hospital of Santiago de Compostela, Santiago de Compostela, Spain; 9 Pharmacy Unit, Gregorio Marañón General University Hospital, Madrid, Spain

**Keywords:** Diseases, Medical research

## Abstract

To analyze the effectiveness of dexamethasone in preventing upper airway obstruction (UAO) symptoms after extubation and the need of reintubation in critically ill children. Multicenter, prospective, double-blind, randomized, phase IV clinical trial involving five pediatric intensive care units. Children between 1 month and 16 years-of-age intubated for more than 48 h were included. Patients were randomized to receive placebo or dexamethasone 0.25 mg/kg every 6 h, 6-to-12 h prior to extubation (four doses). 48 h follow-up was carried out after extubation. Severity of UAO symptoms (Taussig score, stridor) and reintubation requirement were compared. 147 patients were randomized (10 were excluded), 70 patients received dexamethasone and 67 placebo. No global differences were found in the presence of stridor or moderate-to-severe UAO symptoms (Taussig ≥ 5), but Taussig ≥ 5 was less frequent in patients less than 2 years-of-age treated with steroids (*p* = 0.014). Median Taussig score was lower in the dexamethasone group 1 h after extubation, *p* < 0.001. 27 patients required reintubation, 9 due to UAO: 3 (4.3%) in the dexamethasone group and 6 (8.9%) in the placebo group, *p* = 0.319. In those intubated > 5 days, reintubation due to UAO was higher in the placebo group (2.4% vs. 14.3, *p* = 0.052). Nebulized epinephrine and budesonide were required more frequently in the placebo group in the first 2 h (*p* = 0.041) and 1 h (*p* = 0.02) after extubation, respectively. No relevant side effects were observed. Dexamethasone prior to extubation did not significantly reduce moderate-severe UAO symptoms, except for patients under 2-years of age. Dexamethasone could decrease Taussig score and the need of rescue therapies, as well as reintubation rates in those intubated for more than 5 days.

## Introduction

Post-extubation upper airway obstruction (UAO) in children is a common complication in up to a third of patients, thereby increasing morbidity and mortality rates^1–3^. Laryngeal edema is more frequent and severe in children than in adults due to the small diameter of their airways^2,4^. Signs of airway obstruction include stridor and respiratory distress, which may require reintubation in 6–13% of patients^1–3^.

Extubation failure and reintubation has significant effects on the outcomes. It is associated with prolonged length of hospital stay, increase of days of mechanical ventilation and higher healthcare-related costs^1,2,5,6^. For this reason, it is important to prevent this complication, especially in patients at risk. Predisposing risk factors related to post-extubation UAO include underlying respiratory or neurological disease, prolonged intubation (more than 36-48 h), reintubation and young age (≤ 24 months of age)^1,2^.

Dexamethasone and other steroids have been used in an attempt to reduce post-extubation UAO, although with heterogeneous dosing regimens. However, contradictory results have been obtained in studies assessing its effectiveness in reducing UAO symptoms and reintubation rates^5–10^, although recent meta-analysis suggest that the use of steroids could be favorable^11^. In light of the lack of consistent evidence about the effectiveness of steroids in preventing UAO in children, high quality studies are needed.

We designed a randomized study to compare the effectiveness of dexamethasone versus placebo in the prevention of post-extubation UAO in patients intubated for more than 48 h.

The main objective of this study is to analyze the effectiveness of dexamethasone in preventing stridor and reducing the frequency of moderate to severe UAO symptoms in critically ill children compared to placebo. Secondary objectives are to analyze if dexamethasone reduces the incidence of reintubation, the need of treatments for UAO, and to evaluate the adverse effects associated with this treatment.

## Material and methods

A multicenter, prospective, double-blind, randomized, placebo-controlled, phase IV clinical trial was designed (EudraCT 2009-016596-30, registered on 25/08/2010). It was conducted in five Spanish pediatric intensive care units that care for general and cardiac critically ill patients. The study protocol^12^ was developed in accordance with the Standard Protocol (SPIRIT). The study was approved by local Ethics Committee of all participating centers (Fundación para la investigación biosanitaria del Hospital Gregorio Marañón Institutional Review Board, FIBHGM-ECNC003-2010; FIBHGM-10-01). All methods were performed in accordance with the local guidelines and regulations.

### Patients

All patients admitted to the participating pediatric intensive care units (PICUs) between 1 month and 16 years of age who required intubation for more than 48 h were eligible. Exclusion criteria were: (1) airway malformations; (2) suspected or confirmed upper airway infection (croup syndrome, tracheitis or epiglottitis); (3) previous surgeries involving upper or lower airway; (4) administration of steroid therapy within the previous seven days; (5) previous extubation failure during PICU stay; (6) parent refusal to participate in the study. Written informed consent was required from parents or guardians before enrollment.

Sample size was established to detect a 50% reduction in the incidence of UAO according to previously published studies (which is estimated to be around 30%). Approximately 110 subjects were expected to be included in each treatment group^12^.

### Screening, randomization and masking

After identification and enrollment candidates were assigned to one of the two therapy groups on a 1:1 ratio by simple randomization. The coordinating center sent a randomization table of patients to each participating center. The Pharmacy Unit of the coordinating center sent treatment-arm assignments labeled and blinded as established in the randomization table for each center. Study medication (dexamethasone or placebo) was also packed and distributed to each participating center by the Pharmacy Unit of the coordinating center. Medication packages were labeled without any information regarding group assignment. Medication ampoules looked the same in both groups.

### Study intervention

Patients randomized to the treatment group received intravenous dexamethasone 0.25 mg/kg/dose (to a maximum of 8 mg) every 6 h, for a total of 4 doses. Patients randomized to placebo group received normal saline. The first dose was administered between 6 and 12 h prior to extubation. Dose adjustment, discontinuance or restart of treatment was considered a protocol violation.

On inclusion, demographic and clinical data (diagnosis, intubation characteristics and medical history related with upper airway problems) were collected.

The following variables were recorded at 15 min, 1 h, 2 h, 6 h, 12 h, 24 h and 48 h after extubation: (1) UAO modified Taussig score; (2) need for, and frequency of additional therapies used to treat respiratory distress (nebulized epinephrine, nebulized budesonide, iv steroids; based on the clinical criteria of the attending physician); (3) hemodynamic and respiratory parameters; (4) arterial pCO_2_ and pO_2_ and glucose level (blood samples were only drawn if they were needed for clinical purpose); (5) need for reintubation, time point and cause; (6) presence of secondary effects attributable to study treatment: hypertension, hyperglycemia, digestive bleeding and occurrence of infection.

The primary outcome was the reduction in the incidence of moderate to severe UAO symptoms and the reduction of the occurrence of stridor within 48 h after extubation. Moderate UAO symptoms were considered as the presence of stridor or Taussig score ≥ 5. The secondary end points were the need of reintubation and the appearance of adverse effects associated with dexamethasone.

### Statistical analysis

Details about sample size determination are specified in the published study protocol^12^. Continuous variables are presented as mean values and standard deviation (SD), or as median values and interquartile range (IQR), depending on result of Kolmogorov–Smirnov test. Categorical variables are expressed as frequencies and percentages.

Since outcome assessment is not possible if the patient does not undergo extubation, a per-protocol analysis was performed. All patients involved in protocol study violation were excluded from the analysis. The association between categorical variables were assessed by Chi-squared or Fisher test. Continuous variables were compared by Student's *t*-test or Mann–Whitney U test. A linear mixed model was developed to compare the evolution over time of the different variables, considering each individual as a random effect. Follow-up time, dexamethasone treatment and their interaction over time were considered as fixed effects with compound symmetry covariance structure. Linear mixed models estimate fixed and random effects and are especially useful when the dependent variable is binary and involves repeated measures. In a linear mixed model, we obtain a p value indicating the probability of finding a similar behavior of a continuous variable measured repeatedly in two different groups over time. When the comparison between groups in a linear mixed model achieves statistical significance (*p* < 0.05), a different evolution for that variable between both groups over time is considered to be present. Secondly, both groups are compared at each timepoint, obtaining specific *p* values for every timepoint comparison. Linear mixed models can show different evolution of variables over time between groups despite values of variables at each timepoint do not show differences (when variables do not evolve parallel over time). Conversely, linear mixed models can also establish a comparable behavior over time of two variables despite statistically significant differences are present at different timepoints when both variables evolve in parallel form. These models have great flexibility and power to study variables measured repeatedly over time with missing values^13,14^.

A subgroup analysis in children aged younger than one year and two years old and patients with length of intubation of more than five days were additionally performed. Statistical analysis was performed using SPSS version 20 (SPSS Inc, Chicago, USA).

## Results

### Study population

**Figure 1:**
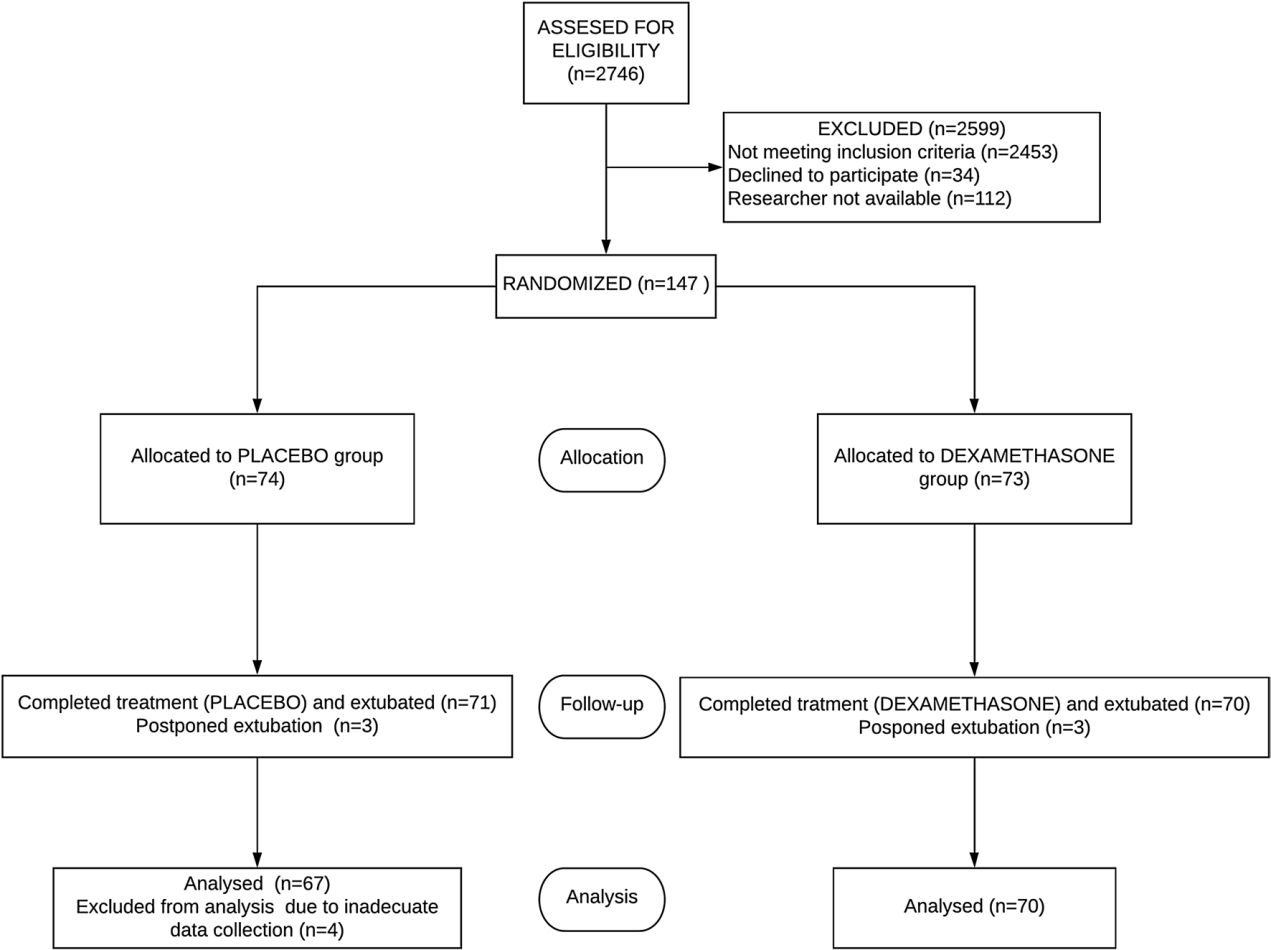
CONSORT flow diagram.

From February of 2013 to February of 2020, 2746 were assessed for eligibility. 293 met the inclusion criteria and 147 patients were randomized (Fig. 1 shows CONSORT flow diagram). There was balance between centers and treatment groups. Ten patients were excluded due to study protocol violations: in 6 children extubation was delayed, in four patients the data collection was inadequate. One hundred and thirty-seven patients (137) were managed per protocol. Seventy (51.1%) received dexamethasone and 67 (48.9%) placebo. Baseline characteristics before extubation are shown in Table 1. There were no statistical differences between groups except for heart rate and blood glucose level prior to extubation.

**Table 1:** Patients’ baseline data prior to extubation (mean and standard deviation, median and interquartile range or number and percentage).

Parameters	Dexamethasone (N = 70)	Placebo (N = 67)	*p*	Test
Weight (kg)	7.3 (4.7–15)	6.7 (4.7–13)	0.646	UMW
Age (months)	7 (2.5–54.0)	9 (4.0–30.0)	0.995	UMW
Length of intubation (days)	8.0 (5.0–13.0)	7.0 (4.0–11.8)	0.308	UMW
Tube size (mm)	4 (4–5)	4 (3.4–4.5)	0.217	UMW
Cuffed tube n/N (%)	56/70 (80%)	57/67 (85.1%)	0.435	CSQ
Oral intubation n/N (%)	59/69 (85.5%)	59/66 (89.4%)	0.496	CSQ
Nasal intubation n/N (%)	10/69 (14.5%)	7/66 (10.6%)		
Tube exchange n/N (%)	16/70 (22.9%)	14/63 (22.2%)	0.930	CSQ
Bronchoscopy requirement previous extubation n/N (%)	5/70 (7.1%)	1/67 (1.5%)	0.209	EFT
Respiratory rate (rpm)	32.0 ± 9.9	35.1 ± 11.3	0.186	TST
Mean BP (mmHg)	74.3 ± 15.1	71.6 ± 14.6	0.298	TST
Heart rate (bpm)	125.8 ± 24.5	136.3 ± 26.0	0.016	TST
Blood glucose level (mg/dl)	130.6 ± 38.5	107.0 ± 24.6	**0.001**	UMW

Chi-squared test (CSQ), Exact Fisher Test (EFT), *T*-student test (TST) or U-Mann Whitney (UMW) test was used. Data regarding intubation approach (oral or nasal) was missed in 2 patients and data regarding requirement of bronchoscopy prior to extubation was missed in 4 patients. Statistically significant values ​​appear in bold.

### Primary and secondary outcomes

**Figure 2:**
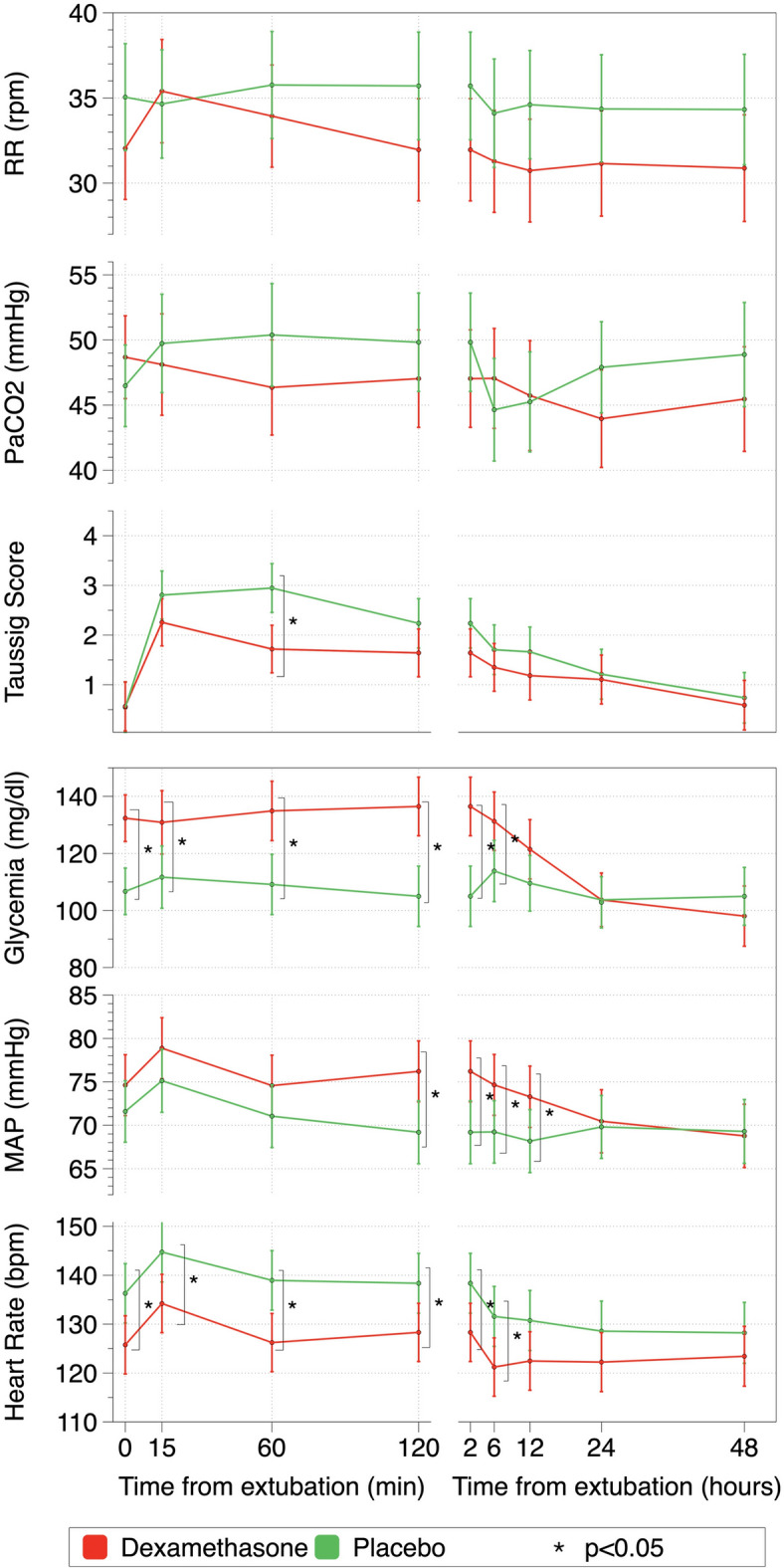
Linear mixed model comparing evolution over time of estimated means and 95% CI after extubation of respiratory (Taussig score, arterial CO2 paCO2 and respiratory rate (RR)), hemodynamic and metabolic parameters (heart rate, mean arterial pressure (MAP) and glycemia). Significant differences in different moments of follow-up (*p* < 0.05) were marked as *.

No differences were found in the presence of stridor or moderate-to-severe UAO symptoms (Taussig ≥ 5) between treatment groups (see Table 2). No differences were found in the maximum Taussig score (registered at any evaluation point) between dexamethasone and placebo group (Table 2). Evolution of Taussig score over time in both treatment groups was compared using a linear mixed model. Global evolution was different between groups (*p* = 0.023, Fig. 2). Additionally, comparison of Taussig scores at the different evaluation timepoints is shown in Table 3. Taussig score 1 h after extubation was lower in the dexamethasone group compared to the placebo group, *p* < 0.001. Evolution over time of the other variables (heart rate, mean arterial pressure, glycemia, pCO_2_, and respiratory rate) introduced in the linear mixed model was comparable between groups except for glycemia (*p* < 0.001, Fig. 2). Individual comparisons at the different timepoints for the different variables are shown in Additional file 3: Table S1.

**Table 2:** Presence of stridor, moderate-severe UAO symptoms, reintubation incidence, reintubation due to UAO and maximum Taussig between treatment groups.

Outcomes		Total cohort (N = 137)	Dexamethasone (N = 70)	Placebo (N = 67)	*p*	Test
Presence of stridor n/N (%)		52/136 (38.2%)	24/69 (34.8%)	28/67 (41.8%)	0.400	CSQ
Subgroup	≤ 2 years (n = 88)	38/88 (43.2%)	16/43 (37.2%)	22/45 (48.9%)	0.269	CSQ
	> 2 years (n = 48)	14/48 (29.2%)	8/26 (29.2%)	6/22 (27.3%)	0.791	CSQ
	> 5 days of intubation (n = 87)	32/87 (36.8%)	14/45 (31.1%)	18/42 (42.9%)	0.256	CSQ
Moderate-to-severe UAO symptoms n/N (%)		40/137 (29.2%)	16/70 (22.9%)	24/67 (35.8%)	0.095	CSQ
Subgroup	≤ 2 years (n = 89)	27/89 (30.3%)	8/44 (18.2%)	19/45 (42.2%)	**0.014**	CSQ
	> 2 years (n = 48)	13/48 (27.1%)	8/26 (30.8%)	5/22 (22.7%)	0.532	CSQ
	> 5 days of intubation (n = 87)	27/87 (31%)	11/45 (24.4%)	16/42 (38.1%)	0.169	CSQ
Reintubation n/N (%)		27 /137 (19.7%)	16/70 (22.9%)	11/67 (16.4%)	0.344	CSQ
Subgroup	≤ 2 years (n = 89)	17/89 (19.1%)	9/44 (20.5%)	8/45 (16%)	0.748	CSQ
	> 2 years (n = 48)	10/48 (20.8%)	7/26 (26.9%)	3/22 (13.6%)	0.259	CSQ
	> 5 days of intubation (n = 87)	22/87 (25.3%)	12/45 (26.7%)	10 (23.8%)	0.759	CSQ
Reintubation due to UAO n/N (%)		9/137 (6.6%)	3/70 (4.3%)	6/67 (9%)	0.319	EFT
Subgroup	≤ 2 years (n = 89)	7/89 (7.4%)	2/44 (4.5%)	5/45 (11.1%)	0.434	EFT
	> 2 years (n = 48)	2/48 (4.2%)	1/26 (3.8%)	1/22 (4.5%)	1	EFT
	> 5 days of intubation (n = 87)	7/87 (8%)	1/45 (2.2%)	6/42 (14.3%)	0.053	EFT
Maximum Taussig score (median, IQR)		3 (1–5)	2 (1–4)	3 (2–5)	0.103	UMW
Subgroup	≤ 2 years (n = 89)	3 (1–5)	3 (1–4)	4 (2–6)	0.080	UMW
	> 2 years (n = 48)	2 (0–4)	2 (0–6)	2 (1–4)	0.675	UMW
	> 5 days of intubation (n = 87)	3 (1–5)	3 (0–5)	3 (2–5)	0.198	UMW

Chi-squared test (CSQ), Exact Fisher Test (EFT), and U-Mann Whitney (UMW), test were used.Chi-squared test (CSQ), Exact Fisher Test (EFT), and U-Mann Whitney (UMW), test were used. Statistically significant values appear in bold.

**Table 3:** Comparison of Taussig Scores between treatment groups at each evaluation timepoint using linear mixed model.

	Dexamethasone		Placebo		p
	Mean	SD	Mean	SD	
Baseline	0.6	0.25	0.5	0.26	0.957
15 min	2.3	0.24	2.8	0.25	0.112
1 h	1.7	0.24	2.9	0.25	**< 0.001**
2 h	1.6	0.24	2.2	0.25	0.091
6 h	1.4	0.24	1.7	0.25	0.314
12 h	1.2	0.25	1.7	0.25	0.179
24 h	1.1	0.25	1.2	0.26	0.765
48 h	0.6	0.25	0.7	0.26	0.681

*T* Student test has been used to compare estimated means. Statistically significant values appear in bold.

Regarding the need of reintubation, 27 patients required reintubation for various reasons, without differences between groups. Nine (33.3%) of these 27 patients were reintubated specifically due to UAO obstruction: 3 (4.3%) in patients treated with dexamethasone and 6 (8.9%) in the placebo group (Table 2).

A mixed model approach was used to analyze the evolution over time after extubation of several respiratory and hemodynamic parameters. No differences were found in the evolution of respiratory rate and paCO2 (*p* = 0.402 and *p* = 0.087 respectively), Fig. 2. Regarding hemodynamic parameters (Fig. 2), the evolution of heart rate (*p* = 0.373) and mean arterial pressure (*p* = 0.077) throughout the follow-up was not different between groups. Nevertheless, mean arterial pressure was higher in the dexamethasone group from 2 to 12 h after extubation (*p* < 0.05) and heart rate was higher in the placebo group from extubation to 6 h of follow-up (*p* < 0.05).

The need for rescue therapies for UAO within 48 h from extubation is shown in Table 4. The use of nebulized epinephrine in the first 2 h after extubation was needed in 16.9% of placebo patients versus 5.8% in the dexamethasone group (*p* = 0.041). Nebulized budesonide was used more frequently in the placebo group, 7.5% versus 0% in the first hour after extubation (*p* = 0.02) and 9.4% versus 1.5% at 12 h after extubation (*p* = 0.05). Intravenous dexamethasone (additional to study medication) in the first hour after extubation was required in 7 patients (10.4%) in the placebo group and in one patient (1.4%) in the dexamethasone group (*p* = 0.03).

**Table 4:** Treatment requirements for UAO symptoms between treatment groups and subgroups within 48 h from extubation.

Treatment requirement		Total cohort (N = 137)	Dexamethasone (N = 70)	Placebo (N = 67)	*p*	Test
Nebulized adrenaline n/N (%)		39/137 (28.5%)	15/70 (21.4%)	24/67 (35.8%)	0.062	CSQ
Subgroup	≤ 2 years (n = 89)	27/89 (30.3%)	9/44 (20.5%)	18/45 (40%)	**0.045**	CSQ
	> 2 years (n = 48)	12/48 (25%)	6/26 (23.1%)	6/22 (27.3%)	0.738	CSQ
	> 5 days of intubation (n = 87)	22/87 (25.3%)	8/45 (17.8%)	14/42 (33.3%)	0.095	CSQ
Nebulized budesonide n/N (%)		12/136 (8.8%)	4/70 (5.7%)	8/66 (12.1%)	0.188	EFT
Subgroup	≤ 2 years (n = 88)	9/88 (11.7%)	3/44 (6.8%)	6/44 (13.6%)	0.484	EFT
	> 2 years (n = 48)	3/48 (6.3%)	1/26 (3.8%)	2 (9.1%)	0.587	EFT
	> 5 days of intubation (n = 86)	9/86 (10.5%)	3/45 (6.7%)	6/41 (14.6%)	0.299	EFT
Corticosteroids (i.v.) n/N (%)		18/137 (13.1%)	7/70 (10%)	11/67 (16.4%)	0.266	CSQ
Subgroup	≤ 2 years (n = 89)	14/89 (15.7%)	5/44 (11.4%)	9/45 (20%)	0.263	CSQ
	> 2 years (n = 48)	4/48 (9.8%)	2/26 (7.7%)	2/22 (9.1%)	1	EFT
	> 5 days of intubation (n = 87)	10/87 (11.5%)	4/45 (8.9%)	6/42 (14.3%)	0.512	EFT

Chi-squared Test (CSQ) and Exact Fisher Test (EFT) test were used. Statistically significant values appear in bold.

Concerning adverse effects, blood glucose level was higher in the dexamethasone group at the baseline measurement and during the first 6 h of follow-up, *p* < 0.05 (Fig. 2), but it decreased and fell to a similar level as in the placebo group at 12 h after extubation. Only one patient, receiving steroids, required insulin therapy due to hyperglycemia. No bleeding, infection, hypertension or other adverse events were observed in the treatment group. PICU length of stay was similar in both groups (23.8 days with dexamethasone vs. 23.4 days with placebo, *p* = 0.906). None of the patients died.

### Subgroups analysis

Analysis of subgroups of higher risk of UAO (patients younger than two years old) and those patients intubated for more than 5 days is shown in Table 2. Moderate-to-severe UAO symptoms were less frequent in patients less 2 years of age treated with steroids (Table 2). There were no global differences in the presence of stridor between treatments in the different subgroups. Nevertheless, the presence of stridor was significantly different at some time-points: twelve hours after extubation it was present in the 20.9% in the control group versus 4.8% in the treatment group in children younger than two years old (*p* = 0.05). In patients with a length of intubation of more than five days, stridor was more frequent in the placebo group one hour after intubation (34.1% vs. 15.6%, *p* = 0.045).

In patients with an intubation of more than five days, reintubation due to UAO obstruction was higher in placebo group than dexamethasone group, with almost significant differences (Table 2).

## Discussion

Intubation and invasive mechanical ventilation are common practices in pediatric intensive care units. The patients requiring intubation are at high risk of presenting complications of the upper airway. UAO symptoms include stridor and respiratory distress, and the most severe consequence is the need of reintubation. Extubation failure has an important impact on prognosis of patients admitted to PICU^2,4^, increasing mortality, length of hospital stay, duration of mechanical ventilation and hospitalization costs^1,2,5,6^.

Corticosteroids, due to their anti-inflammatory effects, have been previously used to prevent UAO, but the results of different studies has been contradictories^5–10^. Most of studies were performed in adults. Steroids reduce incidence of upper airway complications and reintubations in high risk patients in a meta-analysis of adult patients^7^. Studies in pediatric patients are heterogeneous, with small sample sizes, involving different ages, diagnoses, doses and timings of steroids therapy^4,7^.

Our study showed that dexamethasone decreased the UAO severity evaluated using Taussig score over time and the need of rescue treatments for UAO. Moreover, stridor was less frequent in children younger than two years old receiving dexamethasone and in those with prolonged intubation (more than 5 days). Statistically significant decrease of reintubation rates was not reached, although the incidence was the half in patients treated with dexamethasone, but we found a significant reduction in reintubation due to UAO in those intubated for more than 5 days.

Some authors reported a decrease in the incidence of stridor^5,8^, whereas two studies showed a reduction in the incidence of reintubation^8,9,15^. A study in neonates found that dexamethasone reduces the risk for reintubation^10^. In contrast, this benefit has not been proven in other studies in children^16^ and neonates^17^.

There are few randomized double-blind studies performed in children^3,5,8,9,18,19^ (one include adults and children^5^), and our study is one of the largest (see Table 5). Two meta-analysis were published in 2009^4,20^. The review by Khemani et al. concluded that dexamethasone prior to extubation in children reduces the incidence of stridor, but the evidence is insufficient to conclude that rates of re-intubation are reduced^4^. The meta-analysis by McCaffrey et al.^20^ concluded that corticosteroids reduce laryngeal edema and importantly reduce the incidence of extubation failure in critically ill patients of all ages. This study included one more randomized, double-blind study with prednisolone in patients with croup^21^. A posterior study by Baranwal et al.^3^ compared two regimens in high-risk pediatric patients versus placebo: starting dexamethasone therapy 6 h versus 24 h before extubation. The authors found that the 24-h regimen significantly reduced the incidence of UAO, as compared to the 6-h regimen.

**Table 5:** Summary of prospective randomized studies with pediatric patients (not included studies with only neonatal patients).

Author	Year	Type of study	N	Mean/median age	Mechanical ventilation duration	Steroids	Hours from DEX to extubation	Presence of stridor	Score of UAO symptoms	Need of treatment for UAO	Reintubation rate
Tellez et al	1991	Randomized, double-blind, placebo-controlled	153	2.1 ± 0.4 years	72.8 ± 1.1 h	DEX 0.5 mg/kg/6 h, 6 doses	6-12 h	DEX 21% versus CG 29% (*p* > 0.05)		Epinephrine DEX 21% versus CG 29% (*p* > 0.05)	
Baranwall et al	2014	Randomized, double-blind. Two dose groups comparation	124 (66 24 h PD group and 58 6 h PD group)	1.5–2.5 years	8.5 days	DEX 0.5 mg/kg/6 h, 3 versus 6 doses	6 and 24 h (2 groups)	24 h PD 65.2% versus 6 h PD 82.8% *p* = 0.027	Modified Westley DEX reduce *p* < 0.01	Epinephrine 24 h PD 64% versus 6 h PD, 84%), *p* = 0.02	24 h PD 8.2% versus 6 h PD 15.5% (*p* > 0.05)
Anene et al.*	1996	Randomized, double-blind, placebo-controlled	66 (33 each group)	3–4 months	3.3–3.5 days	DEX 0.5 mg/kg/6 h, 6 doses	6–12 h	Lower in DEX group (*p* < 0.05)	Croup score lower in DEX group (*p* < 0.05)	Epinephrine DEX 12.9% versus CG 68.8%, *p* < 0.01	DEX 0% versus CG 21.9%, *p* < 0.01
De Carvalho et al	2020	Randomized, non-double-blind, placebo-controlled	85 (DEX 41, CG 44)	75% < 2 years	5.7–6.5	DEX 1 mg/kg followed by 0.25 mg/kg q 6 h	Variable, 1 mg/kg all + most 1 dose 0.25 mg/kg	DEX 31.8% versus CG 4.9%, *p* < 0.01	Westley lower after 60 min *p* = 0.022	Epinephrine DEX 29.3% versus CG 34.1% *p* > 0.05	DEX 4.5% versus CG 4.9% *p* > 0.05
Malhotra et al	2009	Randomized, double-blind, placebo-controlled	60 (30 children, 30 adults)	7.9 ± 3.9 years		DEX 0.5 mg/kg, 3 doses	4 h before + 6 h and 12 h after extubation	DEX 26.7% versus CG 63.3 *p* < 0.05			DEX 30% versus CG 63.3% *p* < 0.05
Butragueño-Laiseca et al	2021	Randomized, double-blind, placebo-controlled	137 (70 DEX, 67 CG)	2.6 ± 3.7 years	10.3 ± 11.3 days	DEX 0.25 mg/kg, 4 doses	6–12 h before + 12 h after extubation	DEX 34.7%, CG 41.7% *p* = 0.4	Taussig lower in DEX group *p* < 0.05	Epinephrine DEX 5.8% versus CG 16.9%, *p* = 0.04	DEX 4.3% versus CG 8.9%, *p* > 0.05

Acronyms: CG control group DEX dexamethasone.

* This study includes patients with airway diseases.

Our results do not provide enough evidence to recommend systematic administration of dexamethasone prior to extubation in critically ill children, except for intubations of more than 5 days. Our results suggest that dexamethasone reduces UAO symptoms, but the number of patients included in the study was not sufficient to find significant differences in the primary outcomes. Expected sample size (110 patients per treatment arm) could not finally be achieved due to difficulties during recruitment of patients and expiration of study period. Beside this, incidence of UAO in our study population was found to be much lower of previously published studies. This fact implies that the number of patients needed to identify differences between treatment groups would be much higher than initially expected (more than 450 patients per treatment arm). Thus, only a much larger multicenter (and probably international) randomized study could reach this number of patients.

Similar to previous studies^4^ we did not find significant side effects related to the use of corticosteroids. Blood glucose level was slightly higher in the dexamethasone group during the first hours after extubation, but without clinical relevancy. Blood glucose levels were comparable in both groups 12 h after discontinuation of dexamethasone administration. Similarly, baseline heart rate was higher in the dexamethasone-treated group from baseline to the first six hours of follow-up. No other clinically relevant cardiovascular differences were observed. Therefore, corticosteroids would be safe for this indication at the tested dose.

### Limitations

This study has several limitations. Although it was a multicenter, randomized, double-blind study, the number of patients included was not sufficient to reach statistical significance. Some of the inclusion criteria such as previous administration of steroids and anticipation of the first dose 6–12 h before extubation may have hindered the inclusion of a higher number of patients. Furthermore, some patients could not be recruited due to research collaborator availability issues. Besides, the authorized period for the study expired before the estimated sample size was reached. The study excluded high risk patients with croup and upper airway surgery which might affect the generalizability of this study to those populations.

The primary outcome as a clinical score of UAO symptoms could be subjective, in contrast to the objective secondary outcome of extubation failure.

Participants were not homogeneous in age, diagnosis, duration of mechanical ventilation, although both groups were comparable at baseline. However, baseline data were obtained prior to extubation and not prior starting the treatment. We cannot exclude the influence of other factors, as neurologic, cardiac, or respiratory disturbances, on the post-extubation respiratory failure. Although the stratified subgroup analysis by age was planned, the analysis in patients intubated for more than 5 days was post-hoc.

Finally, rescue therapies used to treat UAO symptoms were not protocolized and there could be differences between hospitals and physicians' practices.

## Conclusions

Dexamethasone prior to extubation did not significantly reduce moderate-severe UAO symptoms, except for patients under 2-years of age. There were no differences in stridor. Dexamethasone could decrease Taussig score and the need of rescue therapies. It could also reduce reintubation rates in those intubated for more than 5 days. Further large multicenter studies are needed to assess if corticosteroid could reduce UAO severity and reintubation incidence in critically ill children.

## Supplementary Information

Supplementary Information 1.

Supplementary Information 2.

Supplementary Information 3.
