# Supplementary material for: Multicenter randomized clinical trial comparing dexamethasone versus placebo in preventing upper airway obstruction after extubation in critically ill children
Source: Sci Rep. 2022 Mar 14;12:4336. doi: 10.1038/s41598-022-08178-0 (PMC8921236; doi:10.1038/s41598-022-08178-0)
Supplement: Supplementary file 2 — Supplementary Information 2. [file 41598_2022_8178_MOESM2_ESM.pdf]

CLINICAL TRIAL PROTOCOL

EFFECTIVENESS OF STEROIDS IN PREVENTING UPPER AIRWAY OBSTRUCTION AFTER EXTUBATION IN CRITICALLY-ILL CHILDREN.

Protocol number: FIBHGM-ECNC003-2010(FIBHGM-10-01)

EudraCT: 2009-016596-30

Study phase: Phase IV

Protocol version: V02, July 19, 2010.

Promotor: Gregorio Marañón Health Research Institute.

Authors/Principal Investigator: Dr. Jesús López-Herce Cid. Intensive Care Unit of Gregorio Marañon Hospital.

CONFIDENTIAL The information and data included in this protocol contain privileged or confidential information that is the property of the main author. No person is authorized to make this information public without the written permission of the Main Author. These limitations will also apply to all information considered privileged or confidential that will be provided in the future.

SUMMARY

TITLE

EFFECTIVENESS OF STEROIDS IN PREVENTING UPPER AIRWAY OBSTRUCTION AFTER EXTUBATION IN CRITICALLY-ILL CHILDREN.

PROMOTOR:

Gregorio Marañón Health Research Institute.

PRINCIPAL/MAIN INVESTIGATOR:

Dr. Jesús López-Herce Cid, PhD.

Pediatric Intensive Care Unit, Gregorio Marañón University Hospital. Madrid (Spain).

Gregorio Marañón Health Research Institute, Madrid (Spain).

Mother and Child Health Research Network (Red SAMID).

Department of Public Health and Pediatrics. Complutense University of Madrid (Spain).

POSTAL ADDRESS

Service of Pediatric Intensive Care. Gregorio Marañón General University Hospital. Calle Doctor Castelo 47, 28009. Madrid. Phone: +34-915290329.

ETHICAL COMMITTEE:

Fundación para la investigación biosanitaria del Hospital Gregorio Marañón Institutional Review Board, FIBHGM-ECNC003-2010 (FIBHGM-10-01).

PARTICIPATING CENTERS:

- Hospital General Universitario Gregorio Marañón de Madrid
- Hospital Central de Asturias de Oviedo
- Hospital de Cruces de Baracaldo
- Hospital Valle de Hebrón de Barcelona
- Hospital Clínico Universitario de Santiago de Compostela

Principal investigator	Center
Jesús López-Herce Cid	Hospital General Universitario Gregorio Marañón de Madrid
Corsino Rey Galán	Hospital Central de Asturias de Oviedo
Javier Gil Antón	Hospital de Cruces de Baracaldo
Zuriñé Martinez de Compañón	Hospital Valle de Hebrón de Barcelona
Antonio Rodríguez Núñez	Hospital Clínico Universitario de Santiago de Compostela

Trial registration: EudraCT identifier, 2009-016596-30. Registered on 11 May 2010.

BACKGROUND

Critically-ill pediatric patients frequently require endotracheal intubation. Post-extubation upper airway obstruction (UAO) is a common complication that affects up to a third of patients who require endotracheal intubation [1–3].

Laryngeal edema is more frequent and severe in children than in adults due to the small diameter of their airways. Indeed, this type of edema in children affects the subglottal region more frequently than the glottal region [2,4]. Signs of airway obstruction include stridor and respiratory distress, which require reintubation in 6-13% of patients [1–3].

Risk factors related to post-extubation UAO include underlying respiratory or neurological disease, prolonged intubation (more than 36-48h), reintubation, a young age (≤ 24 months of age), or infants weight less than 5 kilograms [1,2].

The obstruction of the upper airway~~s~~ has significant effects on the evolution of the patient. This complication may increase morbidity and mortality, length of hospital stay, duration of mechanical ventilation and healthcare-related costs, especially when the patient requires reintubation [1,2,5,6].

Steroids are one of the most widely used therapies for post-extubation UAO, as they have anti-inflammatory effects, thereby reducing the risk for laryngeal edema and airway obstruction. A range of steroids such as hydrocortisone, methylprednisolone and dexamethasone have been used for the prevention and management of post-extubation UAO [4,7]. Contradictory results have been obtained in studies assessing the effectiveness of steroids in preventing post-extubation UAO [5–10].

A recent meta-analysis [7] of studies in adult patients with and without UAO risk factors receiving different regimens of steroids showed that steroids reduce the incidence of upper airway complications and reintubation in high-risk patients.

Studies in pediatric patients are based on widely heterogeneous samples of patients, doses and timing of prophylactic therapy ranging from one hour to 24 hours prior to extubation [4,7].

In studies on the use of dexamethasone in children, a variety of regimens have been used versus placebo. Some authors report a decrease in the incidence of stridor [5,8], whereas two studies show a reduction in the incidence of reintubation [8,9]. A study in neonates concluded that dexamethasone reduced the risk for reintubation [10]. In contrast, this benefit has not been proven in other studies in children [11] and neonates [12].

Baranwal et al [3] compared two regimens in high-risk pediatric patients: starting dexamethasone therapy 6 hours vs 24 hours before extubation. The authors found that the 24-h regimen reduced significantly the incidence of UAO, as compared to the 6-h regimen.

A Cochrane's systematic review [4] concluded that the effectiveness of steroids in preventing UAO in children has not been sufficiently demonstrated, and further studies are needed to assess its potential benefits, especially in high-risk patients.

In light of the lack of consistent evidence, we have designed a randomized study to compare the effectiveness of dexamethasone vs placebo in the prevention of post-extubation UAO in high-risk pediatric patients (> 48 hours intubated).

The main objective of this study is to explore whether dexamethasone is effective in preventing and reducing the severity of UAO symptoms in critically-ill children vs. placebo. Secondary objectives include investigating whether dexamethasone reduces the incidence of reintubation and evaluating the potential secondary effects associated with this medication.

METHODS

STUDY DESIGN

This is a multicentric, prospective, double-blind, randomized, placebo-controlled, phase IV clinical trial. Five pediatric intensive care units of hospitals located in Spain will participate in the study. The study protocol was developed in accordance with the Standard Protocol Items: Recommendations for Interventional Trials (SPIRIT) checklist.

ETHICS APPROVAL AND CONSENT TO PARTICIPATE

This study protocol was designed in accordance with the Declaration of Helsinki (1964) and subsequent versions. The final version of this study protocol was approved by Ethics Committee of Gregorio Marañón Hospital, Madrid, Spain (IBHGM-ECNC003-2010) and the local Ethics Committee of all participating centers. The Principal Investigator will ensure that the study is performed in accordance with laws and regulations governing the performance of clinical trials and will communicate protocol modifications to relevant parties.

The rationale for using placebo is that the effectiveness of steroids has not been confirmed to date, and other medications have not been proven to be effective in preventing post-extubation complications.

The study investigator responsible for patient recruitment in each center will provide oral and written information on the study to the patients or their legal representatives when their age or clinical state prevents them from giving consent. Written informed consent will be required prior to inclusion.

The local Ethics Committees will be given access to original records and databases.

STUDY POPULATION

The sample will include children between 1 month and 16 years of age admitted to the ICU who require intubation for more than 48 hours, regardless of their condition. Patients with airway malformations, suspected or confirmed croup syndrome, tracheitis or epiglottitis and those who had previously underwent any surgery involving upper or lower airway will be excluded. Furthermore, other exclusion criteria were the administration of steroid therapy within the last seven days, patients who had a previous extubation failure during the stay or refusal to participate in the study.

RECRUITMENT

Study candidates will be identified by a study physician, who will explain the study to parents or guardians. Written informed consent will be obtained from

parents or guardians prior to inclusion in the study. CONSORT (*Consolidated Standards of Reporting Trials*) flow diagram is shown in Figure 1.

Figure 1 CONSORT Flow Diagram

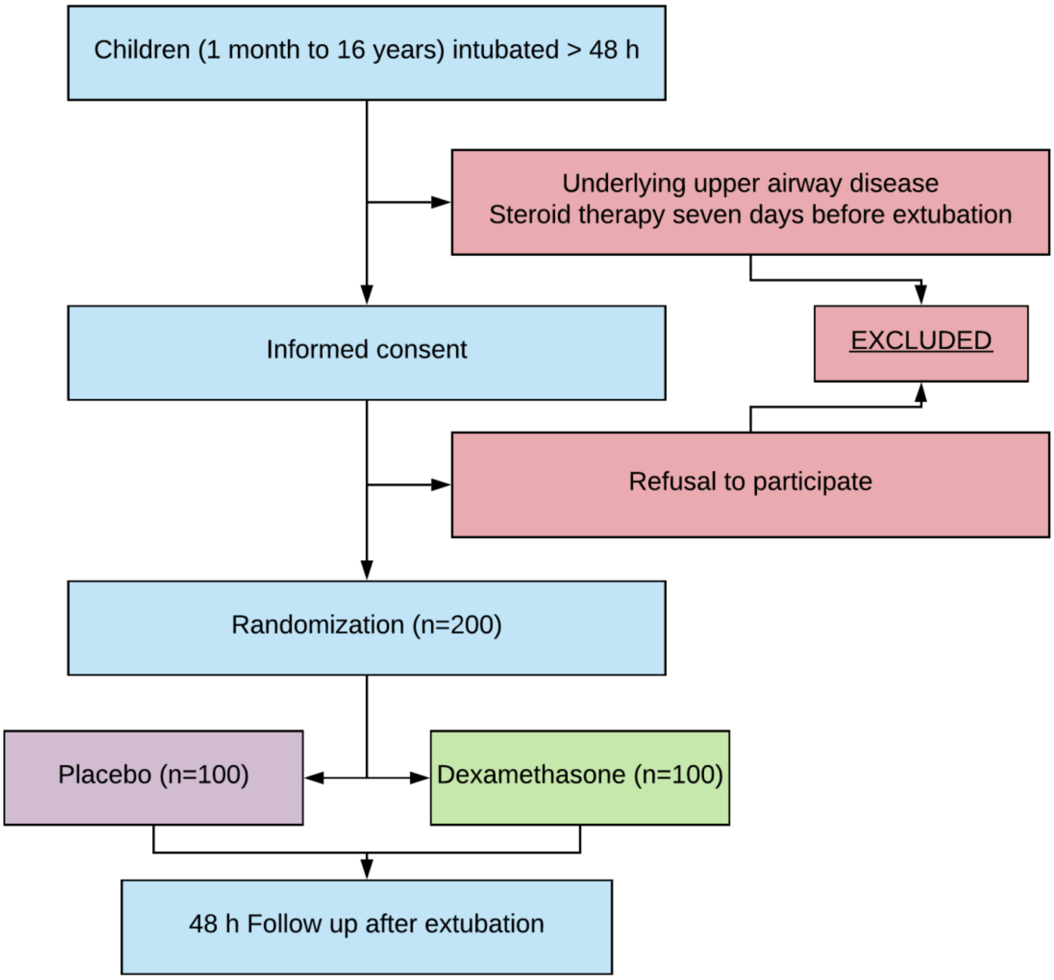

RANDOMIZATION AND MASKING

Patients will be assigned to one of the two therapy groups on a 1:1 ratio by simple randomization using a randomization table on EPIDAT 3.1 (Epidat: software package for epidemiological data analysis. 2006. Consellería de Sanidade, Xunta de Galicia, España; Organización Panamericana de la Salud; Universidad CES, Colombia). The Coordinating Center will send a table for

sequential randomization of patients. This table will contain the number of medication that has been assigned to each patient. The treatment group will not be detailed in the randomization table. This table will also include a number of reserve medication for use in case of deterioration, loss, or if the drug is rendered unusable. The Pharmacy Unit of the Coordinating Center will send treatment-arm assignments labeled and blinded as established in the randomization table for each center.

The labeling and blinding of medication kits will be performed by the Pharmacy Unit of the Coordinating Center, which will keep an open record of these kits and their composition. This record will be coded and stored by the pharmacist of the Coordinating Center responsible for the labeling and submission of samples.

In case of severe or unexpected adverse events related to the medication, the Principal Investigator will require the responsible pharmacist to unblind the codes identifying the sample. The Principal Investigator will be informed so that timely action can be taken. The unblinding of any code will be recorded by the pharmacist responsible for sample blinding.

INTERVENTION

The treatment group will receive intravenous 0.25 mg/kg/dose (to a maximum of 8 mg) every 6 hours, for a total of 4 doses. The first dose will be administered between 6 and 12 hours prior to extubation. The placebo group will be administered saline 0.9%. The study medication will have the same aspect and characteristics than placebo and both will be administered in the same way.

Dose adjustment, discontinuance or reinitiation of treatment will not be allowed. Inspections will be performed to ensure that the treatment is being administered adequately.

DATA COLLECTION AND ASSESSMENT OF EFFICACY

On inclusion, demographic (age, sex and weight) and clinical data will be collected, as follows:

- Diagnosis (six diagnostic groups will be established): surgery, lower airway obstruction, neurological disease, sepsis, trauma and other diagnosis.
- Assessment of severity: the clinical status of the patient will be assessed using PIM2, PRIMS 3 and PELOD scores prior to extubation.
- Size and type of endotracheal tube (cuffed or uncuffed).
- Route of intubation (oral, nasal).
- Previous need for endotracheal tube replacement.
- Previous airway endoscopies.
- Respiratory infection confirmed by endotracheal aspirate culture.
- Presence of blood in endotracheal aspirate.
- Duration of endotracheal intubation prior to extubation (days).

The primary endpoint is the reduction of the incidence of moderate to severe UAO symptoms within 48 hours after extubation. Moderate-severe UAO symptoms will be considered if stridor or Taussig score >5 are present. The secondary endpoints are the presence of reintubation and the potential secondary effects associated with dexamethasone.

A record of the following variables will be kept at 15 minutes, 1h, 2h, 6h, 12h, 24h and 48 hours after extubation:

1. UAO Taussig score[13], a clinical scoring system for the assessment of UAO severity; evaluating stridor, retractions, inflow of air into the lungs, cyanosis and consciousness (table 1). Presence of inspiratory stridor.
2. Need for and frequency of additional therapies for respiratory distress: adrenaline or nebulized budesonide, intravenous steroids, heliox or non-invasive ventilation.
3. Hemodynamic (blood pressure, heart rate) and respiratory (peripheral oxygen saturation, respiratory rate) parameters.
4. Arterial pCO_2 and pO_2 and glycemia. Blood samples will not be routinely collected, they will be drawn only for clinical purpose.
5. Need for reintubation, time point and cause.
6. Presence of digestive bleeding.
7. Occurrence of infection.

The timing of parameter recording is shown in Table 2.

All interventions that patients require for their treatment are allowed but those that could affect the upper airway (respiratory tract infections, reintubations, etc.), will be recorded and will be taken into account in the statistical analysis

A co-investigator will be appointed as responsible for each participating center in order to verify the correct adherence to the study protocol, checking in every 8 hour shift that the medication is prescribed in the treatment and that it has been administered. This investigator will also be responsible of complete follow up of every patient.

Monthly communication newsletter regarding recruitment activity will be sent by email to all researchers. In this newsletter, researchers will also be encouraged to continue recruiting.

Table 1 Modified Taussig score. Upper airway obstruction is considered mild if score is less than 5 points, moderate-severe with ≥5 points. Modified of Taussig LM. Treatment of Laryngotracheobronchitis (Croup). Am J Dis Child 1975;129:790.

		Score			
		0	1	2	3
Clinical parameters	Color	Normal	Normal	Normal	Cyanotic
	Air entry	Normal	Mildly diminished	Moderately diminished	Substantially diminished
	Retractions	None	Mild	Moderate	Severe
	Level of consciousness	Normal	Restlessness if disturbed	Restlessness at rest	Lethargy
	Stridor	None	Mild	Moderate	Severe (or no stridor if severe obstruction)

Table 2. SPIRIT Schedule of Events Timeline: enrolment, assessment of safety, assessment of efficacy, and dispensing of the medicine. Visits 2 to 8, respectively: 15 minutes following extubation, 1 h, 2 h, 6 h, 12 h, 24 h and 48 h after extubation.

Tests	Screening	First visit prior to extubation	Visits 2 to 8
**Assessment of eligibility**			
Informed consent	x		
Inclusion and exclusion criteria	x		
Anamnesis	x		
**Assessment of safety**			
Physical examination	x	x	x
Vital signs	x	x	x
Assessment of adverse events		x	x
Previous and current medications	x	x	x
**Assessment of efficacy** [a]			
Vital signs	x	x	x
Stridor	x	x	x
Taussig scale	x	x	x
Reintubation		x	x
**Other assessments**			
Other medications		x	x
**Deliveries of medication**			
Randomization and dispensing of the medication		x	x
Contact with the randomized-dispensing center	x	x	x

EVALUATION OF SAFETY

The occurrence of adverse events from inclusion to study completion will be recorded for all patients. All patients will be monitored until hospital discharge, including those who discontinue their participation in the study. Severe adverse events during hospital stay will be reported to the Study Coordinator and Promotor. In case of occurrence of severe adverse events, follow-up will be performed until remission or until a diagnosis is made and its association with the study medication is established.

A severe adverse event is defined as any adverse event that the patient's treating physician considers to require treatment on the basis of the characteristics and clinical status of the patient. Severe hyperglycemia is any glycemia > 200 mg/dl that persists for more than 6 hours and/or physicians consider to require treatment on the basis of the clinical status of the patient.

Any unused medication will be disposed in accordance with the protocol established by the Pharmacy Unit in collaboration with the Unit of Environmental Management of the Coordinating center, in compliance with ISO 14.001. Upon study completion, any unused or partially used medication will be sent back to the Coordinating Center.

SAMPLE SIZE

The estimated incidence of moderate-severe UAO symptoms is approximately 33% [1–3]. We consider a clinically relevant result the reduction of moderate to severe UAO symptoms incidence by 50% in the treatment group.

To calculate the minimum sample size, a two-sample two-proportion two-tiled comparison test was used, using arcsine approximation for proportions (Cohen's method). To detect a 50% reduction of incidence (0.33 UAO symptoms proportion in the placebo group, 0.16 expected proportion in the treatment group, i.e. Cohen's h effect size=0.4) with 80% statistical power and 5% significance level, 110 subjects per arm will be needed. A follow-up loss rate of 10% has been estimated.

STATISTICS

Categorical variables will be expressed as frequencies and percentages. Normality of continuous data will be assessed by the Kolmogorov-Smirnov test. Continuous variables will be expressed as mean values and SD, or as median values and interquartile range.

To compare the primary endopoint of outcome between the group receiving dexamethasone and the group receiving placebo Z-test statistics will be used. Per protocol analysis will be performed. An intention-to-treat analysis will be also performed to guarantee the effect of randomization.

The association between other categorical variables will be assessed by Chi-squared test or Pearson's coefficient, or by Fisher's exact test, as appropriate, based on the sample size. Continuous variables will be compared by Student's t-test or Mann-Whitney U test. In comparisons of values with respect to time, Student's t-test of repeated measures or Wilcoxon test will be performed, as appropriate. Additional subgroup analysis according to age and pathologies will be performed.

Bilateral statistical tests will be used at a 5% level of significance. Statistical analysis will be performed using SPSS version 20 (SPSS Inc, Chicago, USA).

DATA PROCESSING AND AUDITING

Data from each patient will be collected and anonymized by a study member in a data log. Each study center will send their data logs to the Coordinating Center. The Principal Investigator will enter data from data logs into a single database for all study patients. An interim analysis will be performed by an independent Data Monitoring Committee (DMC) comprised of members of Central Unit of Clinical Research Support and Clinical Trials (UCAICEC) of the Gregorio Marañón Research Institute. The study will only be interrupted if the incidence of UAO or the incidence of severe adverse events is significantly higher in the steroid treatment group as compared to the placebo group. The final analysis will be carried out when the sample size has been reached.

UCAICEC will be responsible for auditing the trial independently from investigators yearly.

FUNDING

This study was supported by Carlos III Health Institute (Sub-Directorate General for Research Assessment and Promotion and the European Regional Development Fund) as a non-commercial clinical trial (grant number EC11-077).

The study medication and placebo will be provided by Kern-Pharma in their primary packaging as ampoules.

The investigators, patients or their families will not receive any compensation for their participation in this study.

LIST OF ABBREVIATIONS

UAO Upper Airway obstruction

CONSORT Consolidated Standards of Reporting Trials

SPIRIT Standard Protocol Items Recommendations for Interventional Trials

DMC Data Monitoring Committee

REFERENCES

[1] S.C. Kurachek, C.J. Newth, M.W. Quasney, T. Rice, R.C. Sachdeva, N.R. Patel, J. Takano, L. Easterling, M. Scanlon, N. Musa, R.J. Brilli, D. Wells, G.S. Park, S. Penfil, K.G. Bysani, M.A. Nares, L. Lowrie, M. Billow, E. Chiochetti, B. Lindgren, Extubation failure in pediatric intensive care: A multiple-center study of risk factors and outcomes, Crit. Care Med. 31 (2003) 2657–2664. doi:10.1097/01.CCM.0000094228.90557.85.

[2] R.G. Khemani, J. Hotz, R. Morzov, R. Flink, A. Kamerkar, P.A. Ross, C.J.L.L. Newth, Evaluating risk factors for pediatric post-extubation upper airway obstruction using a physiology-based tool, Am. J. Respir. Crit. Care Med. 193 (2016) 198–209. doi:10.1164/rccm.201506-1064OC.

[3] A.K. Baranwal, J.P. Meena, S.C. Singhi, J. Muralidharan, Dexamethasone pretreatment for 24 h versus 6 h for prevention of postextubation airway obstruction in children: A randomized double-blind trial, Intensive Care Med. 40 (2014) 1285–1294. doi:10.1007/s00134-014-

3358-9.

[4] R.G. Khemani, A. Randolph, B. Markovitz, Corticosteroids for the prevention and treatment of post-extubation stridor in neonates, children and adults, Cochrane Database Syst. Rev. (2009). doi:10.1002/14651858.CD001000.pub3.

[5] D. Malhotra, S. Gurcoo, S. Qazi, S. Gupta, Randomized comparative efficacy of dexamethasone to prevent postextubation upper airway complications in children and adults in ICU, Indian J Anaesth. 53 (2009) 442–449. http://www.ncbi.nlm.nih.gov/pubmed/20640206.

[6] T. Fan, G. Wang, B. Mao, Z. Xiong, Y. Zhang, X. Liu, L. Wang, S. Yang, Prophylactic administration of parenteral steroids for preventing airway complications after extubation in adults: Meta-analysis of randomised placebo controlled trials, Bmj. 337 (2008) 1088–1091. doi:10.1136/bmj.a1841.

[7] A. Kuriyama, N. Umakoshi, R. Sun, Prophylactic corticosteroids for prevention of post-extubation stridor and reintubation in adults: a systematic review and meta-analysis, Chest. 151 (2017) 1002–1010. doi:10.1016/j.chest.2017.02.017.

[8] O. Anene, K.L. Meert, H. Uy, P. Simpson, A.P. Sarnaik, Dexamethasone for the prevention of postextubation airway obstruction: a prospective, randomized, double-blind, placebo-controlled trial., Crit. Care Med. 24 (1996) 1666–9. http://www.ncbi.nlm.nih.gov/pubmed/8874303.

[9] D.W. Tellez, A.G. Galvis, S.A. Storgion, H.N. Amer, M. Hoseyni, T.W. Deakers, Dexamethasone in the prevention of postextubation stridor in

children., J. Pediatr. 118 (1991) 289–94. http://www.ncbi.nlm.nih.gov/pubmed/1993963.

[10] R.J. Couser, T.B. Ferrara, B. Falde, K. Johnson, C.G. Schilling, R.E. Hoekstra, Effectiveness of dexamethasone in preventing extubation failure in preterm infants at increased risk for airway edema, J. Pediatr. 121 (1992) 591–596. doi:10.1016/S0022-3476(05)81154-0.

[11] A.F. Saleem, S. Bano, A. Haque, Does prophylactic use of dexamethasone have a role in reducing post extubation stridor and reintubation in children?, Indian J. Pediatr. 76 (2009) 555–557. doi:10.1007/s12098-009-0067-4.

[12] T.B. Ferrara, M.K. Georgieff, J. Ebert, J.B. Fisher, Routine use of dexamethasone for the prevention of postextubation respiratory distress., J. Perinatol. 9 (1989) 287–90. http://www.ncbi.nlm.nih.gov/pubmed/2809781.

[13] L.M. Taussig, Treatment of Laryngotracheobronchitis (Croup), Am. J. Dis. Child. 129 (1975) 790. doi:10.1001/archpedi.1975.02120440016004.
