# Supplementary material for: Multicenter randomized clinical trial comparing dexamethasone versus placebo in preventing upper airway obstruction after extubation in critically ill children
Source: Sci Rep. 2022 Mar 14;12:4336. doi: 10.1038/s41598-022-08178-0 (PMC8921236; doi:10.1038/s41598-022-08178-0)
Supplement: Supplementary file 3 — Supplementary Information 3. [file 41598_2022_8178_MOESM3_ESM.docx]

**SUPPLEMENTARY TABLE 1: Linear mixed model comparing evolution of variables over time between treatment groups. Comparison of global evolution (Fixed effects) and individual timepoints comparisons of estimated marginal means are shown. Statistically significant differences are shown in bold.**

|  |  | PLACEBO | | DEXAMETHASONE | |  |
| --- | --- | --- | --- | --- | --- | --- |
|  |  | Estimated marginal Mean | SD | Estimated marginal Mean | SD | p |
| HEART RATE (bpm) | Fixed effects | - | - | - | - | 0.373 |
|  | Baseline | 136 | 3.1 | 126 | 3.0 | **0.015** |
|  | 15 min | 145 | 3.1 | 134 | 3.0 | **0.016** |
|  | 1h | 139 | 3.1 | 126 | 3.0 | **0.004** |
|  | 2h | 138 | 3.1 | 128 | 3.0 | **0.021** |
|  | 6h | 132 | 3.1 | 121 | 3.0 | **0.018** |
|  | 12h | 131 | 3.1 | 122 | 3.0 | 0.058 |
|  | 24h | 129 | 3.1 | 122 | 3.1 | 0.148 |
|  | 48h | 128 | 3.2 | 123 | 3.1 | 0.28 |
| MEAN ARTERIAL PRESSURE (mmHg) | Fixed effects | - | - | - | - | 0.077 |
|  | Baseline | 72 | 1.8 | 75 | 1.8 | 0.232 |
|  | 15 min | 75 | 1.8 | 79 | 1.8 | 0.145 |
|  | 1h | 71 | 1.8 | 75 | 1.8 | 0.169 |
|  | 2h | 69 | 1.8 | 76 | 1.8 | **0.006** |
|  | 6h | 69 | 1.8 | 75 | 1.8 | **0.035** |
|  | 12h | 68 | 1.8 | 73 | 1.8 | **0.048** |
|  | 24h | 70 | 1.8 | 70 | 1.8 | 0.803 |
|  | 48h | 69 | 1.9 | 69 | 1.9 | 0.845 |
| GLYCEMIA (gr/dl) | Fixed effects | - | - | - | - | **<0.001** |
|  | Baseline | 107 | 4.1 | 132 | 4.1 | **<0.001** |
|  | 15 min | 112 | 5.5 | 131 | 5.6 | **0.016** |
|  | 1h | 109 | 5.4 | 135 | 5.3 | **0.001** |
|  | 2h | 105 | 5.4 | 136 | 5.2 | **<0.001** |
|  | 6h | 114 | 5.5 | 131 | 5.2 | **0.021** |
|  | 12h | 110 | 5.0 | 121 | 5.3 | 0.102 |
|  | 24h | 103 | 4.6 | 104 | 4.8 | 0.898 |
|  | 48h | 105 | 5.2 | 98 | 5.4 | 0.352 |
| TAUSSIG SCORE (Points) | Fixed effects | - | - | - | - | **0.023** |
|  | Baseline | 0.5 | 0.26 | 0.6 | 0.25 | 0.957 |
|  | 15 min | 2.8 | 0.25 | 2.3 | 0.24 | 0.112 |
|  | 1h | 2.9 | 0.25 | 1.7 | 0.24 | <**0.001** |
|  | 2h | 2.2 | 0.25 | 1.6 | 0.24 | 0.091 |
|  | 6h | 1.7 | 0.25 | 1.4 | 0.24 | 0.314 |
|  | 12h | 1.7 | 0.25 | 1.2 | 0.25 | 0.179 |
|  | 24h | 1.2 | 0.26 | 1.1 | 0.25 | 0.765 |
|  | 48h | 0.7 | 0.26 | 0.6 | 0.25 | 0.681 |
| PaCO2 (mmHg) | Fixed effects | - | - | - | - | 0.087 |
|  | Baseline | 46 | 1.6 | 49 | 1.6 | 0.333 |
|  | 15 min | 50 | 1.9 | 48 | 2.0 | 0.557 |
|  | 1h | 50 | 2.0 | 46 | 1.9 | 0.141 |
|  | 2h | 50 | 1.9 | 47 | 1.9 | 0.303 |
|  | 6h | 45 | 2.0 | 47 | 2.0 | 0.39 |
|  | 12h | 45 | 2.0 | 46 | 2.1 | 0.868 |
|  | 24h | 48 | 1.8 | 44 | 1.9 | 0.13 |
|  | 48h | 49 | 2.0 | 45 | 2.0 | 0.236 |
| RESPIRATORY RATE (rpm) | Fixed effects | - | - | - | - | 0.402 |
|  | Baseline | 35 | 1.6 | 32 | 1.5 | 0.174 |
|  | 15 min | 35 | 1.6 | 35 | 1.5 | 0.737 |
|  | 1h | 36 | 1.6 | 34 | 1.5 | 0.408 |
|  | 2h | 36 | 1.6 | 32 | 1.5 | 0.091 |
|  | 6h | 34 | 1.6 | 31 | 1.5 | 0.205 |
|  | 12h | 35 | 1.6 | 31 | 1.5 | 0.083 |
|  | 24h | 34 | 1.6 | 31 | 1.6 | 0.156 |
|  | 48h | 34 | 1.6 | 31 | 1.6 | 0.134 |
